# Enteral tube feeding practices and associated factors among nurses working in South Wollo Zone Specialized and General Hospitals, Wollo, Ethiopia, 2022

**DOI:** 10.3389/fnut.2024.1399651

**Published:** 2024-08-06

**Authors:** Ali Ahmed, Samuel Anteneh, Adem Hussien, Anwar Seid, Yaregal Semanew

**Affiliations:** ^1^Adult Health Nursing, College of Medicine and Health Sciences, Wollo University, Dessie, Ethiopia; ^2^Paediatrics and Child Health Nursing, College of Medicine and Health Sciences, Wollo University, Dessie, Ethiopia

**Keywords:** enteral tube feeding, nurses, practice, associated factors, hospital

## Abstract

**Background:**

Enteral tube feeding is recommended as a route for nutrient delivery in critically ill patients. The practice of enteral tube feeding by nurses significantly influences patient treatment outcomes. Therefore, this study aimed to identify the practices of enteral tube feeding and the associated factors among nurses working in South Wollo Zone Specialized and General Hospitals, Wollo, Ethiopia, 2022.

**Methods:**

A hospital-based cross-sectional study design was conducted on 420 nurses from 1st August to 1st September 2022. Simple random sampling methods were used to select study participants. Data were collected using self-administered questionnaires and an observational checklist. The data were entered into Epi Data version 4.6 and analyzed using SPSS version 26. Variables with a *p*-value <0.05, a 95% confidence interval, and an adjusted odd ratio were considered to be significantly associated with enteral tube feeding practice.

**Result:**

A total of 400 nurses participated in the study, yielding a 95.2% response rate. The overall good practice of enteral tube feeding among nurses was 114 (28.5%) with a 95% CI of 23.8–33. Enteral tube feeding practice was significantly associated with a lack of adequate resources (AOR = 0.359, 95% CI: 0.192–0.673), unfamiliarity with current guidelines (AOR = 0.346, 95% CI: 0.203–0.586), lack of awareness (AOR = 0.511, 95% CI: 0.306–0.673) and the thermal effect of food (AOR = 0.56, 95% CI: 0.348–0.889).

**Conclusion:**

The enteral tube feeding practice among nurses was found to be 28.5%. Significant determinants of enteral tube feeding practice included a lack of awareness, inadequate resources in the institution, and unfamiliarity with the current guidelines for enteral feeding. To improve enteral feeding practices, it is recommended that sufficient resources be provided, guidelines be made readily available, and training programs be conducted for the nursing staff.

## Introduction

### Background

Enteral tube feeding (ETF) involves delivering food directly into the gastrointestinal tract via a tube (1). ETF is effective in improving patients' quality of life (2). The healthcare team, particularly nurses, plays a major role in the management and maintenance of optimal nutritional status in critically ill patients (3).

Global studies in Northeast and Southeast Asia reported that the prevalence of enteral tube feeding-related malnutrition in hospitalized patients is >40% (4). In Korea, the prevalence of malnutrition in hospitalized patients is 22% (5). In India, 22% of participants have poor knowledge of enteral tube feeding, and findings indicate that 78% of samples have poor enteral feeding practices (3).

In African studies conducted at El-Mhala General Hospital in Egypt, it was found that 69.2% of healthcare providers had incompetent practical levels regarding care for neonates with respiratory distress on mechanical ventilation compared to 30.8% who had competent practical levels (6). A study conducted in Ethiopia revealed that 53.8% of nurses had poor enteral tube feeding practices (7).

### Statement of the problem

ETF is a commonly used method for delivering nutrients directly into the gastrointestinal tract of patients who cannot meet their nutritional needs orally. According to the European Society for Parenteral and Enteral Nutrition (ESPEN) and the American Society for Parenteral and Enteral Nutrition (ASPEN) guidelines, ETF is the preferred method for feeding critically ill patients. It is an important means of counteracting the catabolic state induced by severe diseases. These guidelines provide evidence-based recommendations for enteral tube feeding in patients who have a complicated course during their ICU stay, with a particular focus on those who develop a severe inflammatory response (8).

Even though current guidelines are available, studies in Turkey and Saudi Arabia have shown that 128 (65.4%) and 70 (60%) of the participants, respectively, had satisfactory levels of EF practices regarding “Gastrointestinal System (GIS) Tolerance Evaluation.” However, 50.5% of the nurses never paid attention to the amount of product that should be given to the patient at a meal, although they had better practices for evaluating complications (9, 10).

Regarding factors affecting ETF practice, a Jordanian study found that to improve ETF practices, correctly identifying barriers is crucial for developing strategies to overcome them. Key barriers identified included the absence of a feeding tube, delays in physicians' orders, delays in initiating motility agents, a lack of enteral nutrition formula and/or feeding pumps, and delays in the initiation time of enteral feeding. The same study revealed that in critically ill patients, enteral nutrition practices are often based on opinions rather than evidence-based practice (11).

A study conducted in Africa found several key barriers to proper ETF practices. The most significant barrier was the lack of clearly displayed protocols, as mentioned by 69% of respondents. Additionally, 91% of respondents cited a lack of continuous professional development training for nurses as a major limitation. Understaffing was another significant obstacle, with 74% of participants noting an inadequate number of nurses per ward. Finally, 35% of the 113 respondents indicated that insufficient supplies of the necessary enteral feeding tubes made it challenging to adhere to standard protocols (12).

Poor nursing adherence to evidence-based guidelines has significant negative consequences, including higher mortality rates, increased morbidity, delayed recovery, and prolonged hospital stays. Nurses play an intrinsic role in preventing infections, such as aspiration pneumonia and induced obstructions, by adhering to standard guidelines (4).

Nurses play a pivotal role in successfully implementing and monitoring ETF, as their practices and knowledge directly impact patient outcomes. Suboptimal ETF practices can lead to various complications, such as aspiration, diarrhea, and inadequate nutrient intake, which can further compromise the patient's health and prolong their recovery. Therefore, it is essential to understand the factors that influence nurses' ETF practices to identify areas for improvement and ensure the delivery of high-quality nutritional support to patients.

### Significance of the study

This study provides valuable insights into the current state of ETF practices among nurses in the South Wollo Zone of Ethiopia.

Identifying key factors associated with poor ETF feeding practices can guide the development of targeted interventions. Addressing these modifiable barriers has the potential to improve nurses' ETF practices and ultimately benefit critically ill patients.

The study findings contribute to the limited evidence on ETF practices in Ethiopian healthcare. This information can inform policy, education, and training initiatives to strengthen nurses' competencies in this area of patient care.

Improving ETF practices has broader implications for patient outcomes, including reduced malnutrition, better recovery, and enhanced quality of life. The study's recommendations provide a roadmap for healthcare institutions to prioritize and invest in interventions that can lead to these positive patient-centered impacts.

In summary, this study is significant in its ability to identify critical gaps and inform strategies to enhance nurses' enteral tube feeding practices, which is crucial for optimizing the care and outcomes of critically ill patients in the study setting and beyond.

## Literature review

### Enteral tube feeding practice of nurses

Globally, the prevalence of poor ETF-related malnutrition in hospitalized patients has been a significant concern. Studies have reported a wide prevalence range, from 22% in Korea to over 40% in Northeast and Southeast Asia (5, 13). These findings highlight the need for increased attention and improvement in enteral feeding practices across different healthcare settings. Nurses are responsible for ensuring that short-term enteral feeding tubes are placed correctly before using them for gastric emptying, enteral nutrition, or medication administration (14).

Studies conducted in Iran and Saudi Arabia, with sample sizes of 140 and 70 study participants, respectively, revealed that most ICU nurses had inadequate knowledge about enteral feeding. Specifically, 75% of the nurses lacked sufficient knowledge, and 60% had practices that fell below satisfactory levels of ETF (15).

A multi-centered cross-sectional study conducted in China with 560 study participants shows several inconsistencies in ETF practices among nurses. The study found that 36.2% of nurses used the nose-ear-xiphoid method, and 79.5% of nurses used the forehead-xiphoid method to define the internal length of the nasogastric tube (NGT). Most study participants still relied on outdated methods to confirm NGT placement, such as auscultation of injected air (50.2%), bubble test (34.7%), and observing feeding tube aspirate (34.3%).

Bolus feeding was the most commonly used technique for administering ETF. A total of 97.0% of nurses used syringes to measure gastric residual volume (GRV), with 62.7% of them measuring GRV every 4–8 h. The most frequently used GRV threshold values were 200 ml (44.6%) and 150 ml (25.2%). Most nurses stopped feeding immediately when encountering high GRV (84.3%) or diarrhea (45.0%). The nasogastric feeding practices of many clinical nurses were not consistent with international guidelines (16).

The weakest performance among nurses was related to the gavage of 30 cc water, clamping the tube before feeding (1%), and measuring residual stomach content (8%) (17, 18).

Another study conducted in Iran found a significant difference in the time needed to reach the target caloric goal between continuous and intermittent nutrition methods (*p* < 0.05), with the continuous infusion method achieving the target more quickly. The results also showed no significant difference in gastrointestinal (GI) complications between the two groups (*p* > 0.05) (19).

In Africa, few studies have examined EN practices among nurses, and those that have are limited to intensive care unit (ICU) nurses. Despite this, a descriptive research design conducted at the ICUs of Alexandria Main University Hospital and Health Insurance Hospital involved a convenience sample of 60 critical care nurses (CCNs) who provided direct care for adult critically ill patients with enteral feeding. The study found that the majority of CCNs (90%) had poor knowledge, and 88.3% of CCNS had unsatisfactory performance regarding ETF practice.

Several factors, including age, educational level, years of experience, place of work, and knowledge, were found to have statistically significant effects on CCNs' performance in terms of ETF. In contrast, organizational and patient-related factors had no statistically significant effects on CCNs' performance regarding ETF (20).

A descriptive study conducted at Suez Canal University in Egypt with 50 study participants revealed an unsatisfactory level of performance among nurses providing care to patients undergoing enteral tube feeding. Age and experience were correlated with levels of nurse knowledge. However, there were statistically significant differences in practice related to gender, marital status, and graduation (21).

Another study conducted in Egypt indicated that most of the respondents had unsatisfactory enteral tube feeding practices, and there was no statistically significant correlation between demographic characteristics and practice levels (22). In Ethiopia's context, the enteral tube feeding practice level of nurses was found to be 46.2% (7).

### Factors affecting the enteral tube feeding practice of nurses

Despite the availability of numerous verified clinical practice guidelines focusing on enteral feeding for critically ill patients, there remains a wide gap between guideline recommendations and actual nutrition practices. Studies conducted in China in 2018 showed that the frequency of ETF-related training, the presence of full-time ICU nutritionists, hospital level, specific protocols for enteral feeding, and staff position significantly influenced the enteral feeding of ICU patients (23).

In clinical practice, nutrition, including enteral nutrition (EN), is often not prioritized, leading to risks and safety issues for patients and healthcare professionals. Over 62 studies have identified various patient risks associated with ETF, including the lack of clinical assessment, inadequate tube management, missing energy targets, absence of nutritionists, poor hygiene and handling, incorrect time management and speed, nutritional interruptions, incorrect body positioning, gastrointestinal complications, infections, missing or non-adherence to guidelines, understaffing, and lack of education (24).

A recent study conducted at Saveetha Medical College and Hospital found that among 50 samples, 22 (44%) had mild complications, 18 (36%) had moderate complications, and 10 (20%) had severe complications associated with nasogastric tube feeding (25).

In a 2020 study conducted in Turkey, improper enteral feeding resulted in abdominal distension (28.8%) and gastrointestinal diarrhea (44.2%). There were no reports of aspiration, but 1.9% of vomiting cases were noted. Additionally, among the metabolic problems reported were hyponatremia (30.8%), hypokalemia (42.3%), and hypoalbuminemia (96.2%). Of the patients receiving enteral feedings, 46.2% experienced hyperglycemia, and the most common mechanical problem was tube dislocation (26).

A study in Kampala found that nurses exhibited a poor attitude toward ETF practices. The majority, 75 (62.5%), felt uncomfortable when inserting a nasogastric tube, 92 (76.7%) would not accept the insertion of a nasogastric tube if they were ill, and 72 (60%) believed that all patients felt uncomfortable during the insertion process (27).

A multicenter cross-sectional study conducted in Jordan with 131 study participants identified insufficient nursing staff to deliver adequate nutrition (60%) and fear of adverse events due to aggressive feeding (56%) as the most significant barriers (11). A literature review conducted in 2020 indicated that the most common barriers to nutrition delivery included inadequate resourcing, a lack of nutrition protocols, and gastrointestinal intolerance. The identified facilitators included nutrition education and a supportive multidisciplinary team (28).

A retrospective study conducted in the United States showed that the use of a nurse-led, evidence-based feeding protocol significantly increased nutritional intake by 90%. In contrast, protocol misuse led to only 34% of nutritional intake (29).

According to a descriptive study conducted in Iran with 170 respondents, nurses' knowledge of enteral feeding regarding residual volume (88, 82%), pulmonary aspiration (61, 76%), and tube care (56, 47%) was found to be below the intended level. However, ICU nurses had more knowledge about enteral tube feeding compared to those working in other medical areas (30).

A retrospective multicenter literature review demonstrated that patients who received NGT feeding experienced more complications than those who received precautious endoscopic gastrostomy (PEG) feeding. A higher percentage of PEG patients (47.5%) had no complications compared to the NGT group (8.3%). NGT patients were more likely to experience tube blockage (95%), secondary displacement of the tube (95%), and accidental tube removal (OR 0.03, 95%) (13).

In Brazil, a qualitative study showed that poor enteral feeding practices led to various risks related to the tube, diet, contamination, and routine procedures (31). A quantitative study in India using 100 staff nurses selected through a convenient sampling technique revealed that the majority of them had average knowledge (54%) and fair practices (58%) concerning nasogastric tube feeding. There was no significant association with age, gender, educational qualification, total work experience, the present area of work, duration of work in the present area, or in-service education program attendance related to nasogastric tube feeding at a *p*-value of <0.05 (32).

A study conducted in Kenya indicated that the majority of the respondents had knowledge of nutrition guidelines, the existence of nutrition protocols in the ICU, the preferred route of enteral nutrition, and the initiation time within 24–48 h of ICU admission. However, the amount of gastric residual volume for the next feed to be withheld varied among the respondents (33).

A comparative study in Egypt demonstrated a strong correlation between the utilization of ultrasound for assessing gastric residual volume, nasogastric tube positioning, and standard protocol methods, suggesting that ultrasound is a safe, simple, and effective practice for ICU nurses (34).

A study conducted at Cairo University Hospital with 40 respondents showed that nurses had a satisfactory level of knowledge and an unsatisfactory level of practice but a positive attitude regarding nasogastric tube feeding among critically ill patients. There was a statistically significant relationship between knowledge, practice, and attitude level, with significant differences based on years of experience (35).

Similarly, a quantitative descriptive cross-sectional design was conducted in Kigali, Rwanda, with 69 nurses selected using non-probability convenience sampling from ICU and emergency departments. The results demonstrated that the most predominant perceived barrier by nurses was feeding interruption (95.7%), followed by the lack of feeding formula (95%), while the least predominant barrier was the lack of training on enteral feeding (68%), followed by the lack of guidelines (78.3%), which contradicts other research results (36).

According to a study conducted in South Africa, out of 207 study participants, more than two-thirds (75.4%) of the participants considered themselves competent to administer enteral nutrition, and 29.3% reported having protocols in their workplace (29.3%), with 79.6% referring to these protocols once or twice per month. The most common sources of nutrition knowledge were in-service training (24.9%) and nursing college (20.6%). Participants preferred lectures provided by dietitians (45.4%) to upgrade their nutrition knowledge. No significant differences were found between knowledge and professional rank or between knowledge and years of working experience (37).

An Ethiopian study that solely examined the practices of ICU nurses without addressing the crucial variable of “attitude” found that 53.8% of nurses had poor enteral nutrition practices, and 67.7% had inadequate understanding. Holders of a bachelor's degree were less likely to be informed. The age of nurses, their training in enteral nutrition, and the existence of guidelines and protocols for enteral feeding procedures in ICUs were found to have a substantial impact on the enteral nutrition practices of nurses.

## Objectives

### General objective

To assess enteral tube feeding practices and associated factors among nurses working in South Wollo Zone Specialized and general hospitals, Wollo, Ethiopia, in 2022.

### Specific objectives

- To determine enteral tube feeding practices among nurses working in South Wollo Zone Specialized and general hospitals from 1st August to 1st September 2022.
- To identify factors affecting enteral tube feeding practices among nurses working in south Wollo Zone Specialized and general hospitals from 1st August to 1st September 2022.

## Methods and materials

### Study area

The study was conducted in the South Wollo zone of the Amhara Regional State, located in northeast Ethiopia, 520 km from Bahir Dar and 401 km from Addis Ababa. According to the 2007 census, the total population of South Wollo was 2,518,862, with 11.98% of the population being urban inhabitants. The largest ethnic group reported in South Wollo is the Amhara, making up 94.33% of the population.

In this zone, there is one comprehensive specialized hospital, Dessie Comprehensive Specialized Hospital, which employs 390 nursing staff. Additionally, there are four general hospitals: Boru-Meda General Hospital (80 nurses), Kombolcha General Hospital (56 nurses), Akesta General Hospital (63 nurses), and Mekaneselam General Hospital (72 nurses). The total number of nurses in those hospitals is 504.

### Study design and period

The hospital-based cross-sectional study design was conducted from 1st August to 1st September 2022.

### Population

#### Source population

All nurses working in the South Wollo Zone Specialized and general hospitals.

#### Study population

All ward nurses working in the South Wollo Zone Specialized and general hospitals who were also available during the data collection period.

### Inclusion and exclusion

#### Inclusion criteria

Ward nurses who are working full-time and presented during the data collection period.

#### Exclusion criteria

Nurses working in the outpatient department.

Student nurses who were on practical attachment

### Sample size determination

The sample size was calculated using the single population proportion formula, taking a 95% confidence interval, a 5% margin of error, and the proportion (*p*) from a similar study (53.8) (7). Then, the sample size was determined as follows:


$$\begin{array}{l} \;n=\;\left(z\alpha /2\right)x^{2}\times p\left(1-p\right)/\left(d\right)2\frac{\;}{}\\ =\;\left(1.96\right)2^{*}0538\cdot \left(1-0.538\right)/\left(0.05\right)2=382\\ \text{ add }10\%\text{ non}-\text{response rate }n=420 \end{array}$$

The sample size calculated from the second objective was less than the first objective. Therefore, the final sample size was determined to be 420.

### Sampling techniques and procedures

**Figure 1:**
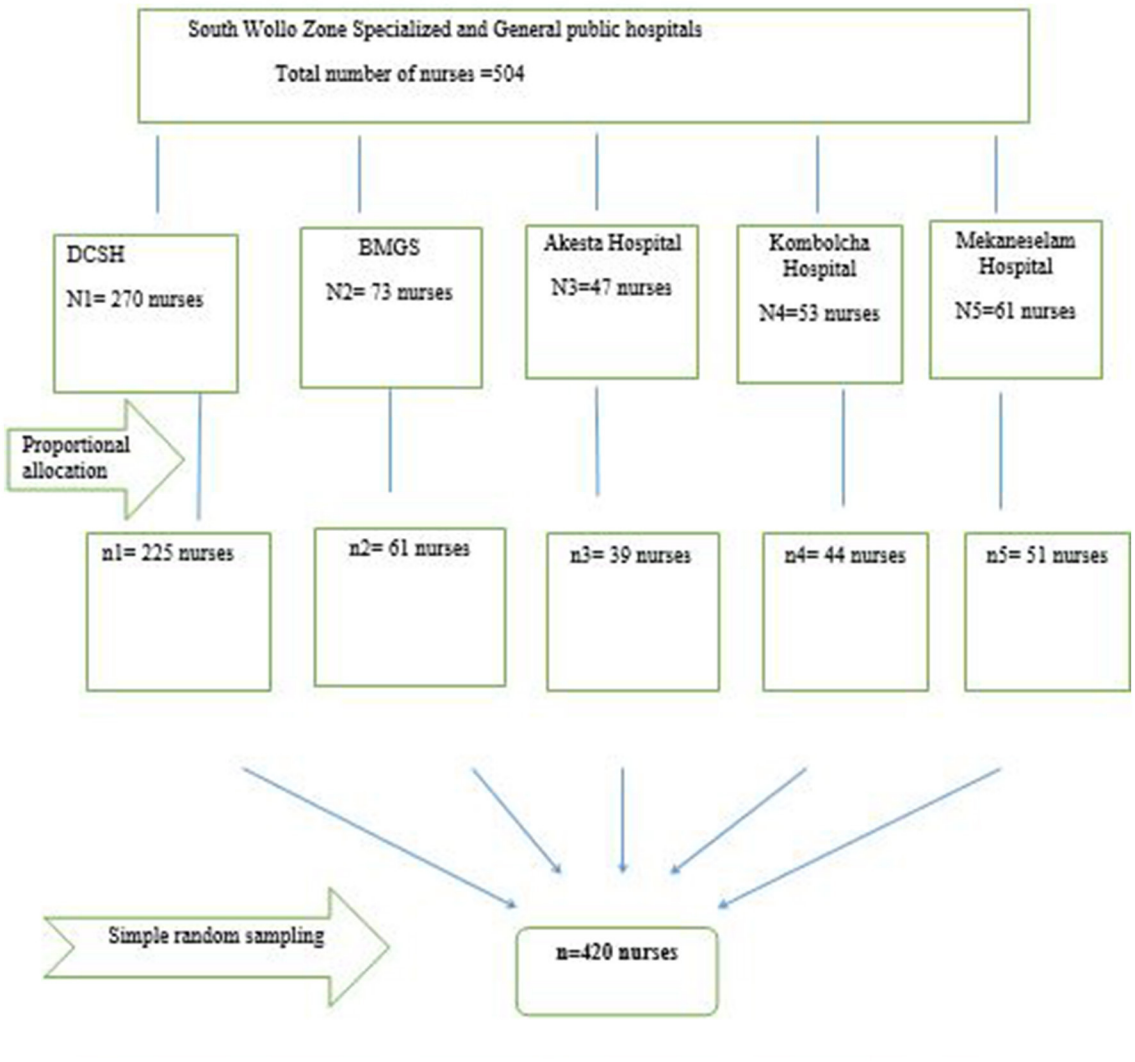
Sampling procedure of enteral tube feeding practice associated factors among nurses working in South Wollo Zone Specialized and General Hospitals, Wollo, Ethiopia, 2022.

Proportional allocation was made for each hospital based on the number of nurses in each hospital, and simple random sampling methods were then used to select study participants from each hospital (Figure 1).

### Dependent variables

The practice of nurses toward ETF.

### Independent variables

Nurse-related factors: nurses' knowledge and nurses' attitudes.

Socio-demographic factors: age, sex, level of education, in-service training, and working experience.

Patient-related factors: resource availability, awareness of patients and their attendants about ETF, participation in decision-making toward enteral tube feeding of the hospitalized patient, and complexity of patient cases such as intolerance and aggressiveness.

Human resources and material-related factors: availability of guidelines and protocols, nurse workforce, ultrasound, x-ray, and stethoscope.

### Operational definition

Enteral tube feeding: the delivery of nutritionally complete feed via a tube into the gut, used for patients who cannot meet their nutritional needs orally (38).

Adequate knowledge: participants scored above the mean of the given questions.

Inadequate knowledge: participants scored below the mean of the given questions (21).

Good practice: participants scored >70% on practice questions.

Poor practice: participants scored <70% on practice questions (35).

Positive attitude: participants scored >80% on attitude questions.

Negative attitude: participants scored >80% on attitude questions (39).

Lack of awareness by the patients and attendants: the nurse explains the procedure in detail, but the patients and their attendants do not cooperate with tube feeding.

Inadequate resources: there is no tube in place, no functional stethoscope, no feeding bag, no feed formula, and no different-size syringes.

Unfamiliarity with current guidelines: participants do not review the guidelines at least once a month and do not use the displayed protocols.

### Data collection tools and procedure

Data were collected using a self-administered, structured questionnaire. The questionnaire was initially prepared in the English language based on the study objective, focusing on the background information of ETF. To ensure consistency, it was translated into Amharic and then translated back into English. The questionnaire was adapted from different kinds of literature (4, 7, 36). Self-administered questionnaires were used to gather socio-demographic and factor variables, whereas a 4-point Likert scale questionnaire and an observational checklist were used to assess practice-related aspects.

The data were collected by five data collectors with diploma qualifications and two supervisors with BSc degrees in nursing. The data collection tools included five sections: socio-demographic characteristics (sex, age, educational level, working experience), knowledge, attitude, practice, and associated factors.

- Knowledge assessment: fourteen (14) questions were used. A score of “1” was given for correct answers and “0” for incorrect answers.
- Attitude assessment: ten (10) questions were included, with four response options: strongly disagree, disagree, agree, and strongly agree. A value of “1” was given for agree and strongly agree, while strongly disagree and disagree were coded as “0.”
- Practice assessment: sixteen (16) dichotomous choice questions (done and not done) were used. “Done” was given a “1” point, and “not done” was given a “0” point.
- Factor variables: these had dichotomous options (yes and no). A “1” point was given for “yes” (indicating a challenge) and a “0” point for “no.”

The data collectors provided the self-administered structured questionnaire to study participants to gather information on enteral tube feeding practices and associated factors. They also used an observational checklist to assess the practical aspects.

### Data quality assurance and control

The supervisors and data collectors underwent a 1-day training program before the actual data collection. The training covered introducing themselves, explaining the study objectives, understanding the tools, sampling techniques, interviewing, and administering the questionnaire in both English and Amharic.

To maintain the tool's consistency and feasibility, a pre-test was conducted 7 days before the actual data collection period using a 5% sample at Kemise General Hospital. The Cronbach's alpha test results were as follows: knowledge = 0.735, attitude = 0.766, and practice = 0.753, indicating that the tool was reliable. Additionally, the tools were reviewed by experts.

The correct implementation of the data collection procedure, completeness, and logical consistency were supervised daily by the supervisors and principal investigator. The principal investigator also carefully checked the data during entry and thoroughly cleaned it before starting the analysis.

### Data processing and analysis

The collected data were checked, coded, cleaned, and entered into Epi Data version 4.6 and then exported to SPSS version 26 for analysis. Multicollinearity was assessed using the variance inflation factor (VIF), with all values being <10. Model fitness was checked using the Hosmer and Lemeshow test, which resulted in a value of 0.717 (71.7%). Bivariate and multivariate logistic regression methods were used to identify associations between dependent and independent variables. All independent variables from the bivariate logistic regression model with a *p*-value <0.25 were included in the multivariable logistic regression model to control for possible confounding effects. Variables with a *p*-value of <0.05 at a 95% CI and an adjusted odd ratio were considered to have a significant association with enteral tube feeding practice. Descriptive statistics, such as frequency and percentage, are presented in texts, tables, and charts.

### Ethical consideration

Ethical clearance was obtained from the Ethical Review Committee of Wollo University, College of Medicine and Health Sciences, Department of Adult Health Nursing (RCSPCS/27/14). Official letters were submitted to the concerned hospital authority to obtain permission, and permission letters were acquired from the quality office. The purpose, benefits, and risks of the study were explained to the study participants. In addition, they were assured of their right to withdraw from the study at any time. The estimated time to complete the questionnaire was communicated to them. Verbal consent was obtained from all study participants, and confidentiality was assured.

## Result

### Socio-demographic characteristics

A total of 400 study participants participated in the study, resulting in a 95.2% response rate. Of the total participants, 228 (57%) were men, with ages ranging from 20 to 54 years and a mean age of 32.42 (SD ± 5.13) years. Regarding educational status, the majority of the study participants, 304 (76), were BSc holders. Additionally, 326 (81.5%) had <10 years of experience, and 325 (81.3%) had received school training (Table 1).

**Table 1:** Socio-demographic characteristics of Nurses in South Wollo Zones Specialized and General Hospitals Wollo, Ethiopia 2022 (*N* = 400).

**Variables**	**Cases**	
	**Number**	**%**
**Sex**		
Male	228	57
Female	172	43.
**Age (years)**		
21–30	181	45.3
31–40	199	49.8
41–50	18	4.5
51–60	2	0.5
**Educational status**		
Diploma	67	16.8
BSC	304	76
MSC	29	7.2
**Year of experience**		
<10 years	326	81.5
11–20 years	68	17
21–30	2	0.5
31–40 years	4	1

### The practice of nurses toward enteral tube feeding

According to this current study, the result showed that only 28.5% (114) of the participants had good practice in enteral tube feeding (95% CI: 23.8–33). The majority of nurses, 279 (69.8%), did not explain the procedure to the patient, and 234 (58.5%) of them did not wash their hands during the procedure. Additionally, 259 of them (64.8%) did not return the residual volume or flush the NG tube with water before and after feeding.

However, some practices were correctly followed by the majority of the nurses. Specifically, 367 (91.7%) of the nurses assembled the equipment before the procedures, 268 (67%) of them positioned the patient in a semi-Fowler's position, and 313 of them (78.2%) correctly confirmed the position of the tube in the stomach (Table 2).

**Table 2:** The practice of nurses toward enteral tube feeding practice in South Wollo Zones Specialized and General Hospitals, Ethiopia 2022 (*N* = 400).

**Variables**	**Number**	**%**
**Wash hands**		
Done	165	41.5
Not done	234	58.5
**Assemble equipment**		
Done	367	91.7
Not done	33	8.3
**Explain the procedure**		
Done	121	30.2
Not done	279	69.8
**Position the patient in the semi-Fowler's position**		
Done	268	67
Not done	132	33
**Provide privacy**		
Done	98	24.5
Not done	302	75.5
**Confirm the position of the tube in the stomach**		
Yes	313	78.2
No	87	21.8
**Aspirate to check the residual volume**		
Done	188	47
Not done	212	53
**Return the residual volume and flush the NG tube with water**		
Done	141	35.2
Not done	259	64.8
**Clamp the NG tube**		
Done	191	47.8
Not done	209	52.2
**Give intermittent tube feeding**		
Done	220	55
Not done	180	45
**Give continuous tube feeding**		
Done	204	51
Not done	196	49
**Provide nasal and oral hygiene**		
Done	156	39
Not done	244	61
**Position the patient for comfort**		
Done	196	49
Not done	204	51
**Document relevant information**		
Done	173	43.3
Not done	227	56.7
**Report any problems**		
Done	206	51.5
Not done	194	48.5

### Nurse-related factors

Respondents appeared to be knowledgeable about enteral tube feeding practice, with 204 of them (51%) scoring more than the mean. Specifically, 326 (81.5%) of the respondents clearly understood the types of enteral tube feeding. The majority, 289 (72.3%), correctly identified when enteral tube feeding should start, and 239 (59.8%) of them were familiar with the preferred feeding method in their ward.

However, more than half, 226 (56.5%), did not correctly identify whether the GI tube was in the appropriate place, and 225 of them (56.3%) did not correctly answer the maximum time to reach the target feeding volume. This may be supported by the variable response, with 267 of the respondents (66.8%) reporting that they were unaware of the availability of any guidelines and 276 (69%) of them stating that there was no protocol for enteral tube feeding in their ward.

Based on the findings of this study, the majority of the respondents, 314 (78.5%; 95% CI: 74.3–84), had a negative attitude toward enteral tube feeding practice. Among the total respondents, 226 of them (56.5%) believed that enteral tube feeding is complicated to administer, 195 (48.8%) thought it was time-consuming due to increased recording, and 223 (55.8%) felt that the procedure was unnecessary.

A high prevalence of negative attitudes was found in responses to attitude measuring items, with participants believing and agreeing that enteral tube feeding practice increases work overload, hospital stay, and medical expenses. Specifically, 241 (60.3%) thought it increased treatment costs, 228 (57%) believed it extended hospital stays, and 229 (57.3%) felt it increased work overload (Table 3).

**Table 3:** Frequency of nurses' attitudes toward enteral tube feeding practice in South Wollo Zone Specialized and General Hospitals in Wollo, Ethiopia, 2022.

**Items for measuring attitude toward enteral tube feeding practice**	**Strongly disagree**	**Disagree**	**Agree**	**Strongly agree**
	**No (%)**	**No (%)**	**No (%)**	**No (%)**
Decrease hospital stay	22 (5.5)	78 (19.5)	228 (57)	72 (18)
Reduce the cost of treatment	29 (9.3)	95 (23.8)	229 (57.3)	47 (11.8)
Minimal time to record	21 (5.3)	195 (48.8)	160 (40)	24 (6)
Easy to administer	62 (15.5)	226 (56.5)	91 (22.8)	21 (5.3)
Is necessary	108 (27)	223 (55.8)	61 (15.3)	3 (2)
Decrease workload	28 (7)	105 (26.3)	241 (60.3)	26 (6.5)
I prefer enteral tube feeding as the first feeding option for ill patients	125 (31.3)	156 (39)	99 (24.8)	20 (5)
Nursing professionals need to care for a patient	151 (37.8)	187 (46.8)	46 (11.4)	16 (4)
Easy procedure	39 (9.8)	195 (48.8)	148 (33)	18 (4.5)

### Resources and patient-related factors

Based on the analysis of resource and patient-related factors, 283 (70.9%) of the respondents reported that there was poor participation of patients or their attendants in decision-making regarding enteral tube feeding. Additionally, 259 (63.5%) of them responded that some patients or guardians refused enteral tube feeding. The majority of them, 301 (75.3), reported a lack of awareness among patients and attendants about enteral tube feeding. Furthermore, 322 of the respondents (80.5%) were unfamiliar with the current guidelines for enteral tube feeding, and 255 of them (63.5%) were still following outdated feeding protocols. A significant number of respondents, 293 (73.3%), believed that there was a work overload (Table 4).

**Table 4:** Resources and patient-related factors toward enteral tube feeding practice in South Wollo Zones Specialized and General Hospitals, Ethiopia 2022 (*N* = 400).

**Variables**	**Number**	**%**
**Lack of awareness by patients and attendants**		
Yes	304	76
No	96	24
**Inadequate resources**		
Yes	95	23.8
No	305	76.3
**Poor participation of the patient and their attendants**		
Yes	283	70.9
No	117	29.3
**Patients' or guardians' refusal of enteral tube feeding**		
Yes	254	63.5
No	146	36.5
**Patient case complexity**		
Yes	300	75
No	100	25
**Physicians order delays**		
Yes	113	28.3
No	287	71.7
**Work overload**		
Yes	293	73.3
No	107	26.8
**Unfamiliarity with current guidelines**		
Yes	148	37
No	252	63
**Following outdated feeding protocols**		
Yes	146	36.5
No	254	63.5
**Interruption during diagnosis and management**		
Yes	129	32.3
No	271	67.8
**Thermic effects of food during enteral feeding**		
Yes	189	47.3
Nom	211	52.7

### Factors associated with nurses' enteral tube feeding practice

In the bi-variable analysis, several factors were identified as determinants of ETF practices. These include the following:

- Lack of awareness by the patient and their attendant.
- Inadequate resources in the institution.
- Thermic effect of the food.
- Feeding interruption during diagnostic and management procedures.
- Physicians' delays in ordering enteral feeding.
- Knowledge of nurses.
- Attitude of nurses.
- Patients' or guardians' refusal of enteral feeding.
- Unfamiliarity with the current guidelines for enteral feeding.

Several factors were identified as significant determinants of enteral feeding practice in the multivariable binary logistic analysis. These included lack of awareness, inadequate resources in the institution, the thermic effect of the food, and unfamiliarity with the current guidelines for enteral feeding.

- Inadequate resources: the presence of inadequate resources was associated with a 0.36 times lower likelihood of good enteral tube feeding practice compared to those with adequate resources (AOR = 0.36, 95% CI: 0.19–0.67).
- Unfamiliarity with guidelines: Nurses unfamiliar with the current guidelines were 0.35 times less likely to practice good enteral tube feeding compared to those familiar with the guidelines (AOR = 0.35, 95% CI: 0.2–0.59).
- Thermal effect of food: the thermal effect of food reduced the likelihood of good enteral tube feeding practices by 44% compared to commercially prepared foods (AOR = 0.56, 95% CI: 0.35–0.89; Table 5).

**Table 5:** Bi-variable and multivariable logistic analysis of determinants of enteral feeding practice in South Wollo Zones Specialized and General Hospitals, Ethiopia 2022 (N = 400).

**Variables**	**Poor practice**	**Good practice**	**Bi-variable analysis**		**Multivariable analysis**	
			**COR**	**95%CI**	**AOR**	**95% CI**
**Lack of awarenes**						
Yes	229 (80.1%)	75 (65.8%)	0.479	(0.295, 0.776)	0.511	(0.306, 0.854)^*^
No	57 (19.9%)	39 (34.2%)	1		1	
**Inadequate resource**						
Yes	80 (28%)	15 (13.2%)	1	(0.214, 0.712)	1	
No	206 (72%)	99 (86.8%)	0.39		0.359	(0.192, 0.673)^*^
**Thermic effect of the food**						
Yes	145 (50.7%)	44 (38.6%)	0.611	(0.393, 0.952)	0.56	(0.348, 0.899)
No	141 (49.3%)	70 (61.4%)	1		1	
**Feeding interruption by procedure**						
Yes	98 (34.3%)	31 (27.2%)	0.716	(0.444, 1.157)	0.882	(0.511, 1.520)
No	188 (65.7%)	83 (72.8%)	1		1	
**Physicians' delays**						
Yes	86 (30.1%)	27 (23.7%)	0.722	(0.438, 1.190)	0.874	(0.510, 1.499)
No	200 (69.9%)	87 (76.3%)	1		1	
**Unfamiliarity with current guidelines**						
Yes	126 (44.1%)	22 (19.3%)	0.304	(0.180, 0.511)	0.343	(0.201, 0.585)^*^
No	160 (55.9%)	92 (80.77%)	1		1	
**Knowledge of nurses**						
Yes	146 (51%)	50 (43.9%)	0.749	(0.484, 1.159)	0.906	(0.568, 1.446)
No	140 (49%)	64 (56.1%)	1		1	
**Attitude of nurses**						
Yes	68 (23.8%)	18 (15.8%)	0.601	(0.339, 1.065)	0.707	(0.383, 0.862)
No	218 (76.2%)	96 (84.2%)	1		1	
**Patients or guardian's refusal**						
Yes	185 (64.7%)	69 (60.5%)	0.837	(0.535, 1.309)	1.069	(0.648, 1.764)
No	101 (35.3%)	45 (39.5%)	1		1	

COR^*^, crude odds ratio at a p-value of <0.25; AOR^**^, adjusted odds ratio at a p-value of <0.05.

## Discussion

This study aimed to identify the magnitude of nurses' ETF practice and its associated factors.

This study's findings revealed that 28.5% (95% CI: 23.8–33) of nurses had good practice in enteral feeding. Significant factors influencing ETF practice included lack of awareness, inadequate resources within the institution, the thermic effect of food, and unfamiliarity with current ETF guidelines.

These findings are consistent with a descriptive study conducted involving a sample of 68 nurses working in the ICU at El-Mhala General Hospital in Egypt, where 30.8% of participants had good practice (6). However, the findings of these studies are more promising than those of the study conducted at Alexandria University Hospital in Egypt, where only 11.7% (30) of the nurses practice good ETF. The possible difference was that they used a minimal sample size of 60, which may lead to an ecological fallacy.

Conversely, the findings of this study are less promising than those from studies conducted in Japan (70%) (15, 40), Iran (57%) (17), Saudi Arabia (40%) (15), and Addis Ababa, Ethiopia, where 46.2% of nurses had good ETF practice (7). A correlational study conducted in Iran may differ from this one in terms of methodology. A sample size difference could also exist, with 140 nurses from Iran, 70 nurses from Saudi Arabia, and 196 nurses from Addis Ababa. Finally, the study was conducted in all wards, whereas the Iranian study was conducted in an ICU.

The study's conclusions included a gap in the practice of enteral feeding and factors such as insufficient funding, ignorance of guidelines, lack of awareness, attitude, knowledge, physician delays, patient or guardian refusal, and feeding interruptions during procedures. Even though half of the respondents (51%) stated that they knew about enteral tube feeding, this knowledge was insufficient for excellent enteral tube feeding practices (27).

A high prevalence of negative attitudes was found in attitude-measuring items, with participants believing and agreeing that enteral tube feeding practice increases work overload and prolongs hospital stays and medical expenses. Specifically, 60.3% (241 participants) believed it increased treatment costs, 57% (228 participants) thought it lengthened hospital stays, and 57.3% (229 participants) felt it added to the work overload.

The findings of this study showed that 41.5% of nurses (95% CI: 36.5–46.2) had good hand washing practices, and 35.3% of them (95% CI: 30.5–40.7) had good practices of flushing the tube before and after feeding. These findings are consistent with a study in Saudi Arabia, which reported 44% and 31% (10), respectively. Regarding knowledge of enteral tube feeding practices, 51% (95% CI: 45.5–56.3) of the study participants had good knowledge. This finding is more promising than the results from the study conducted in Iran at 25% (15) and 11.2% (30), Egypt at 10% (30), and Addis Ababa, Ethiopia, at 33.3% (7). The difference may be due to variations in study design, sample size, and study setting.

### Factors associated with enteral tube feeding practice

The lack of adequate resources, unfamiliarity with current guidelines, lack of awareness, and thermal effects were significantly and negatively associated with enteral tube feeding practices. Those with inadequate resources were 64% less likely to practice good enteral tube feeding than those with adequate resources (AOR = 0.36, 95% CI: 0.19–0.67). These findings are consistent with the study conducted in Addis Ababa, Ethiopia, which reported an AOR of 0.38 (7). Similarly, these findings are supported by a study conducted in Jordan (11), indicating that the resource issue is not only patient related but also hospital related.

Additionally, the study found that those unfamiliar with current guidelines were 65% less likely to engage in good enteral tube feeding practices than those familiar with the guidelines (AOR= 0.35, 95% CI: 0.20–0.59). Moreover, patients and their attendants who lacked awareness were 49% less likely to practice good enteral tube feeding compared to those who were aware (AOR= 0.51, 95% CI: 031–0.85).

These findings highlight that inadequate resources, lack of awareness, and unfamiliarity with guidelines are interconnected issues. Insufficient resources may have a direct impact on the availability of guidelines, leading to unfamiliarity and a lack of awareness about enteral tube feeding practices. Based on these studies, it was also found that 69.8% of nurses did not explain the procedure to the patient.

Similarly, 70.9 % of patients and their attendants demonstrated poor participation in patient care, and 75% of the cases were complicated by patient-related factors. Delivering enteral tube feeding care requires ongoing education and training. This study revealed that the majority of nurses (81.5%) involved in tube feeding practice had only received training during their schooling and had never received in-service training on tube feeding. This is in direct conflict with guidelines, which state that all healthcare professionals involved in enteral tube feeding should be oriented to standardized practices, as the absence of such guidelines may negatively affect the quality of care.

The fourth associated factor revealed that the thermal effect decreases good enteral tube feeding practice by 44% compared to using commercially prepared foods (AOR = 0.56, 95% CI: 0.35–0.89). Feeding hot fluids through the enteral tube immediately after preparation is indeed challenging. Consequently, nurses may not wait until the fluid cools to a lukewarm temperature before feeding the patient, leading to guardians being obliged to feed the patients themselves, which can significantly impact enteral tube feeding practice. These findings are inconsistent with those of a study conducted in Rwanda, which reported a rate of 13% (36).

### Strengths of the study

- Broad study area: the study was conducted in five hospitals, covering a large geographic area, which enhances the generalizability of the findings despite the large sample size.
- Novel findings: the study provides new insights, serving as a valuable springboard for future researchers in the field.
- Limited prior research: With only one previous study on enteral tube feeding in Ethiopia, this research fills a significant gap and offers additional insights into enteral tube feeding practices in the region.

### Limitations of the study

The practice-measuring items were filled out through observation, which may have caused participants to be more conscious, perform the tasks more carefully, and, as usual, potentially introduce bias.

## Conclusion and recommendations

### Conclusion

The lack of awareness, inadequate institutional resources, thermal effects of food, and unfamiliarity with current enteral feeding guidelines were significant determinants of poor enteral tube feeding practice among nurses. The authors recommend providing sufficient resources and training on current enteral feeding guidelines.

### Recommendation

Based on the findings of this study, the following recommendations are made:

**To nurses**

- Update their knowledge and skills by reading protocols and guidelines in books and on websites.
- Attend onsite training and engage in practical sessions.

**To hospital administrators and nurse leaders**

- Empower and encourage nurses to update their knowledge and practice using enteral feeding protocols and guidelines to improve their practice.
- Enhance health education about enteral tube feeding practice, ensuring that nurses explain the indication and importance of enteral tube feeding before the procedure.
- Provide guidelines and onsite training.
- Ensure the availability of equipment, materials, and supplies needed for enteral tube feeding practice and make fees as affordable as possible for those who cannot afford them.
- Advise patients who can afford it to buy commercially prepared foods.

**To Amhara Regional Health Bureau**

- Update nutritional guidelines and distribute them in a timely manner.
- Initiate training programs.
- Conduct regular follow-ups and supervision.
- Work with stakeholders to obtain free support for commercially prepared foods, training, and so on.
- Create awareness about nutrition through mass media.

**To the Ministry of Health, Ethiopia**

- Develop guidelines and protocols for healthcare settings with input from multidisciplinary teams, including nurses.
- Provide a strategic plan for the implementation of enteral feeding practices in various service areas that care for critically ill patients.

**To researchers**

- Conduct research using various research designs on the practice of enteral feeding for critically ill patients at the national level.

## Data availability statement

The raw data supporting the conclusions of this article will be made available by the authors, without undue reservation.

## Ethics statement

The studies involving humans were approved by College of Medicine and Health Sciences, Wollo University, Ethical Review Committee. The studies were conducted in accordance with the local legislation and institutional requirements. The participants provided their written informed consent to participate in this study.

## Author contributions

AA: Conceptualization, Data curation, Formal analysis, Funding acquisition, Investigation, Methodology, Project administration, Resources, Software, Supervision, Validation, Visualization, Writing – original draft, Writing – review & editing. SA: Conceptualization, Supervision, Visualization, Writing – review & editing. AH: Conceptualization, Methodology, Supervision, Writing – review & editing. AS: Conceptualization, Visualization, Writing – review & editing. YS: Visualization, Writing – review & editing.
